# Antioxidant Function of Steen Solution

**DOI:** 10.1155/2014/578353

**Published:** 2014-11-23

**Authors:** Mohamed S. A. Mohamed

**Affiliations:** Thoracic Transplantation Department, University Clinic Essen, Hufeland Straße 55, 45147 Essen, Germany

This is a comment on “New insights into the Steen solution properties: breakthrough in antioxidant effects via NOX2 downregulation” [1].

The addressed paper investigated reactive oxygen species (ROS) production and NADPH oxidase (NOX2) activity in platelets, polymorphonuclear leukocytes, and lymphocytes-monocytes, while being incubated in buffer solution and in Steen solution. The results showed significant role of Steen solution to reduce ROS production and to downregulate NADPH oxidase activity [1]. However, treatment with apocynin resulted in more significant downregulation of NOX2 and ROS production [1].

During ex vivo lung perfusion (EVLP), antagonizing ROS production would be important for the attenuation of cytokine production, which may constitute the stimulus for subsequent graft dysfunction and rejection after transplantation (the level of IL8 correlates positively with the development of primary graft dysfunction; meanwhile, the level of IL6 correlates positively with the 30-day mortality) [2].

During graft ischemia, the absence of flow would be translated into membrane depolarization and increased ROS production, with associated increased NOX2 activity and inhibition of K_ATP_ channels [2]. The increased ROS are involved in a signaling cascade that leads to increased expression of adhesion molecules on endothelial cells, which are essential for the attachment and migration of inflammatory cells [2].

To antagonize ROS during EVLP, antioxidants or NOX2 inhibitors might be used. However, caution should be taken with that because EVLP might be considered as an ischemic preconditioning model. This hypothesis is supported by the observation that lung grafts are tolerant to both cold and normothermic post-EVLP ischemia [3]. In such scenario, ROS would be important for graft conditioning for the subsequent ischemic period between EVLP and transplantation (the presence of antioxidants during the preconditioning phase was found to diminish the degree of protection) [4].

Accordingly, the use of Steen solution with its antioxidant potential and its inhibitory effect on NOX2 during the graft cold static preservation phase would be expected to result in a better outcome than that of the currently applied protocols.

In addition, the presence of albumin and dextran in Steen solution would provide further protection for the graft during cold preservation. Furthermore, supplementation of Steen solution with K^+^ channel agonists would have a dual benefit through decreasing ROS production during initial ischemia and graft conditioning and protection during the subsequent ischemic-reperfusion injury [2, 5].

Formerly this year, a new EVLP protocol (Shehata protocol) was introduced by the author which recommended the use of Steen solution (supplemented with antioxidants and K^+^ channel agonists) during cold static graft preservation as well as EVLP [6]. The results of the addressed paper are supportive of this recommendation.
